# On Shifting Percussion Dulness

**Published:** 1905-03-18

**Authors:** 


### Hospital Clinics.

### ON SHIFTING PERCUSSION DULNESS.

In a lecture on this subject in relation to certain surgical diseases Mr. Rickman J. Godlee[1] has made a contribution of considerable importance to all interested in the physical examination of the chest and abdomen. He has re-investigated the influence of position upon areas of dulness found in various parts of the trunk, and has come to certain conclusions which are directly opposed to current teaching and which have an intimate bearing on practical diagnosis and treatment. Taking first the question of dulness over the base of one or other lung, Mr. Godlee argues that when such dulness shifts on placing the patient on his face or upon the opposite side, this fact usually means that the pleura is healthy and the cause of the dulness is sub-diaphragmatic—that the disease, in short, is not above but below the diaphragm. A parallel statement to this is that in cases of inflammatory effusions into the chest the amount of movement of the dulness on changing the position of the body is so small as to be almost a negligible quantity; in a localised empyema without gas in the cavity there is no change, and even in a serous effusion the change, if any, is of the slightest. On the other hand, shifting dulness over the back of the chest may be due, as already explained, to a collection of fluid—for example, a sub-diaphragmatic abscess—or to a solid growth immediately below the diaphragm; further, it must be remembered that the liver dulness behind is always diminished (unless there are pleural adhesions) when the patient is placed on his face or in the knee-elbow position, this being specially true if at the same time the patient takes a full inspiration, when, indeed, the dulness may almost or altogether disappear. These last facts suggest a general review of the facts of percussion in relation to the position of the patient; and in order to conduct this Mr. Godlee had made a high table with a net top so that the under-surface of the body could be examined in any position of recumbency. In a healthy young male it is found that in the erect position there is very little resonance over the abdomen; it is often impossible to define the lower border of the liver with anything like certainty, and the lower abdomen and left flank are nearly always more or less dull. The cæcum, the transverse colon, and the lateral aspect of the stomach are usually the only areas of tympanitic resonance. In the prone position the dulness becomes more extensive, and may include the whole of the anterior surface, or may leave some resonance over the transverse colon and cæcum. The dorsal position permits definition of the lower border of the liver, and gives resonance over the small intestines and the anterior surface of the stomach, whilst the gastric resonance in the axillary area is replaced by dulness. The explanation of these facts, doubtless, is that in a healthy young adult with good digestion there is not much gas in the small intestine, and only a moderate amount in the stomach and colon; the fluid or semi-solid contents gravitate downwards and cause dulness over the front of the gastric area and lower abdomen. The dulness of the spleen and left kidney are greatest in dorsal decumbency, and least with the patient on his face or in the knee-elbow position. These facts, and the diminution of the posterior area of hepatic dulness in the face position, emphasise the conclusion that dulness which shifts with position is the normal condition of both pulmonary bases behind, and is no proof of fluid in one or other pleural cavity.

Regarding abdominal conditions associated with varying dulness, it is known that in cases of intestinal obstruction with much distension the flanks may be dull when the patient is lying on his back, but the uppermost may become resonant when he is placed on his side—and this without free fluid in the peritoneal cavity. The suggested explanation is that in an obstruction of the large intestine the fluid contents of the intestine gravitate to the dependent parts and the gas rises; whilst in an obstruction of the small intestine coils of the bowel loaded with fluid contents sag over and replace those distended with gas. In cases of ascites, again, the so-called typical horse-shoe dulness is often absent, the most frequent deviation being the presence of resonance in the right inguinal region, due presumably to the distension of the cæcum with gas. The real test of a general ascites is that the dulness in changing the position of the body shifts with great rapidity, whereas in localised collections of fluid such change occurs very slowly or not at all. Another point in ascites is that often a more extensive area of dulness is obtained when the finger is pressed lightly on the abdominal wall than when it is pressed more firmly; in the latter case the pressure removes a thin layer of fluid which, with light pressure, intervenes between the air-filled viscera and the abdominal wall. A similar experience may be met with in enlargement of the liver, where a coil of intestine has passed on to its anterior surface; here the more firm pressure displaces the bowel and gives a greater area of dulness. Unless this is remembered, the tympanitic percussion over the hepatic area might lead to the conclusion that free gas was present in the peritoneal cavity. Another possible fallacy is the disappearance of the anterior hepatic dulness in distension of the abdomen from intestinal obstruction. This is explained by the tilting up of the liver so that only a limited part of its convex surface lies against the anterior abdominal wall. Hence absence of the hepatic dulness is not a trustworthy sign of gas in the peritoneal cavity. In rupture of a gastric or intestinal ulcer no doubt both gas and fluid are present in the peritoneal cavity, but such cases are nowadays usually operated on—or else the patient dies—before the characteristic physical signs develop. But in exceptional instances a condition develops in which there is formed a huge cavity, containing gas and pus, bounded posteriorly by the abdominal viscera matted together by lymph, and in front by the abdominal and thoracic parietes. The gas may press the diaphragm upwards, and thus the physical signs of pneumothorax may be suggested. But care will show that the tympanitic note and bell-sound are continuous and the same over the whole of the cavity, that the liver dulness has disappeared, and that when the patient is turned on his side some part of the tympanitic area instantly becomes dull. A localised peritoneal abscess containing gas may be met with in appendicitis, and the "lump" detected in an early examination may later seem to have disaappeared, being concealed by gaseous distension, and may thus give a false appearance of improvement.

Another important fact in regard to percussion dulness, both in the abdomen and chest, is that it is often diminished when the patient is anæsthetised. In the abdomen, as for example in the case of an abscess or inflammatory exudation around the appendix, the muscular contraction which is involuntarily produced as a protection to the inflamed parts pushes away neighbouring coils of intestine; under the anæsthetic the muscles relax and the intestinal coils are able more or less to slip back to their natural position, and thus to establish a tympanitic area. The diminution of the dulness under the influence of an anæsthetic in the case of the chest is less easy to explain, but it must mean a more free movement of the lung, so that a greater expansion of this occurs in the neighbourhood of the previously dull area; in an empyema disappearance of the dulness may indicate a pyo-pneumothorax, the change in the position of the patient having brought the gas from the upper part of the cavity into the field of operation.

[1] The Lancet, February 25, 1905.

